# Assessment of androgen receptor expression in breast cancer patients using 18 F-FDG PET/CT radiomics and clinicopathological characteristics

**DOI:** 10.1186/s12880-023-01052-z

**Published:** 2023-07-17

**Authors:** Tongtong Jia, Qingfu Lv, Bin Zhang, Chunjing Yu, Shibiao Sang, Shengming Deng

**Affiliations:** 1 Department of Nuclear Medicine, The First Affiliated Hospital of Soochow University, Suzhou, 215006 China; 2 Department of General Surgery, The First Affiliated Hospital of Soochow University, Suzhou, 215006 China; 3 Department of Nuclear Medicine, Affiliated Hospital of Jiangnan University, Wuxi, 214122 China

**Keywords:** Breast cancer, Androgen receptor, Radiomics, ^18^F-FDG PET/CT, Clinicopathological, Machine learning

## Abstract

**Objective:**

In the present study, we mainly aimed to predict the expression of androgen receptor (AR) in breast cancer (BC) patients by combing radiomic features and clinicopathological factors in a non-invasive machine learning way.

**Materials and methods:**

A total of 48 BC patients, who were initially diagnosed by ^18^F-FDG PET/CT, were retrospectively enrolled in this study. LIFEx software was used to extract radiomic features based on PET and CT data. The most useful predictive features were selected by the LASSO (least absolute shrinkage and selection operator) regression and t-test. Radiomic signatures and clinicopathologic characteristics were incorporated to develop a prediction model using multivariable logistic regression analysis. The receiver operating characteristic (ROC) curve, Hosmer-Lemeshow (H-L) test, and decision curve analysis (DCA) were conducted to assess the predictive efficiency of the model.

**Results:**

In the univariate analysis, the metabolic tumor volume (MTV) was significantly correlated with the expression of AR in BC patients (p < 0.05). However, there only existed feeble correlations between estrogen receptor (ER), progesterone receptor (PR), and AR status (p = 0.127, p = 0.061, respectively). Based on the binary logistic regression method, MTV, SHAPE_Sphericity_CT_ (CT Sphericity from SHAPE), and GLCM_Contrast_CT_ (CT Contrast from grey-level co-occurrence matrix) were included in the prediction model for AR expression. Among them, GLCM_Contrast_CT_ was an independent predictor of AR status (OR = 9.00, p = 0.018). The area under the curve (AUC) of ROC in this model was 0.832. The p-value of the H-L test was beyond 0.05.

**Conclusions:**

A prediction model combining radiomic features and clinicopathological characteristics could be a promising approach to predict the expression of AR and noninvasively screen the BC patients who could benefit from anti-AR regimens.

## Introduction

Breast cancer (BC) has already become the most frequently diagnosed tumor and the leading cause of cancer-related death among women worldwide [1, 2]. According to the different expressions of specific molecular receptors, BC can be classified into various subtypes, which are tightly associated with the treatment strategy selection and prognosis [3, 4]. However, even with classic targeted therapies, a certain proportion of patients are insensitive to treatments and develop varying degrees of resistance [5–8]. Therefore, more attention should be paid to new molecular targets to assist in the diagnosis and treatment of BC.

Most recent studies have investigated the roles of androgen receptor (AR) in BC patients, showing that AR has different subtypes with considerable levels of expression [9, 10]. AR and estrogen receptor (ER) competitively bind to estrogen-dependent signaling pathways, and this crosstalk may contribute to acquired resistance to endocrine therapy [11, 12]. Besides, the expression of AR is highly correlated with the amplification of human epidermal growth factor 2 (HER2), which is coupled with AR activation and enhances the oncogenic function of certain signaling pathways [13, 14]. Triple-negative BC (TNBC) lacks recognized molecular targets, in which the expression of AR is related to ER-regulated gene transcription and a lower rate of pathological complete response (pCR) to neoadjuvant chemotherapy [15, 16]. Consequently, early recognition of AR expression not only helps predict prognosis but also guides the application of AR-targeted therapy, effectively improving patients’ sensitivity to traditional therapies and prolonging their overall survival.

At present, the expressions of molecular receptors are generally detected through immunohistochemistry (IHC). Such an approach is mainly based on biopsy as an invasive procedure, and it is rarely repeatable and unrepresentative due to small tissue samples and intratumor heterogeneity [17]. ^18^F-FDG PET/CT (Fluorine-18 fluorodeoxyglucose positron emission tomography/computed tomography), which combines functional metabolic quantification and morphological imaging, is commonly used for initial staging, post-treatment assessment and re-staging in BC [18, 19]. Radiomics, which uses medical imaging to noninvasively quantify intratumoral heterogeneity, has emerged as a translational research topic in BC [20]. Recent literature has indicated that radiomic features obtained from ^18^F-FDG PET/CT can contribute to the prediction of tumor molecular subtypes, while there are still limited studies focusing on the role of AR as a predictive parameter [17].

In the present study, we aimed to develop a machine-learning model for individual prediction of AR expression in BC patients by exploring the correlations between radiomic features, clinicopathological factors, and AR status. In addition, our findings might help identify the patients who could benefit from the AR-targeted treatment.

## Materials and methods

### Patients

This retrospective study was approved by the institutional review board of the First Affiliated Hospital of Soochow University, and the informed consent was waived because of its retrospective nature. Our current study was conducted in accordance with the Declaration of Helsinki, and the trial registration number of this study was ChiCTR2200062858.

All patients were histologically diagnosed as primary BC and underwent ^18^F-FDG PET/CT imaging for staging between December 2017 and April 2022. The specific inclusion criteria were as follows: (1) aged beyond 18 years; (2) without any therapy before the standard examination of ^18^F-FDG PET/CT; (3) with histological data (type and grade) derived from biopsy (generally for large lesions) or surgical specimens (routinely for relatively smaller lesions); (4) with IHC examination of AR performance; and (5) with complete clinical and imaging datasets. Exclusion criteria were as follows: (1) without complete ^18^F-FDG PET/CT images or good imaging quality; (2) with the too-small size of the primary lesion for segmentation; (3) with multiple lesions of BC; (4) with other types of cancers; and (5) with unclear histological proof.

### ^18^F-FDG PET/CT imaging

The patients were injected ^18^F-FDG (4.07–5.55 MBq/kg) for routine preparation after fasting for at least 6 h to ensure the blood glucose level was below 11.1 mmol/L. Approximately 40–60 min later, an integrated PET/CT scanner (Discovery STE, General Electric Medical Systems, Milwaukee, WI, USA) was adopted to acquire images from the base of the skull to the midthigh. With the acquisition parameters (transaxial field of view of 70 cm, pitch of 1.75, rotation time of 0.8 s, and slice thickness of 3.75 mm), low-dose (140 kV, 120 mA) CT images were acquired for the following attenuation correction and anatomic localization. Immediately, PET image acquisition was performed for 2–3 min per bed position. Finally, the ordered subset expectation maximization algorithm was used for image reconstruction.

### Pathological evaluation

Histological sections stained with hematoxylin and eosin (H&E staining) were observed under the microscope to confirm the histologic type and grade by two pathologists independently. The expressions of ER, PR, HER2, Ki-67, and AR were detected by IHC. More than 10% of tumor nuclei with positive AR staining were defined as AR positive (AR+) regardless of staining intensity, otherwise defined as AR negative (AR-). As for ER and PR, both proportional staining (%) and staining intensity (weak, medium, and strong) were considered. The combination of IHC scores (1+, 2+, and 3+) and fluorescence in situ hybridization (FISH) was used to determine HER2 status. Ki-67 was divided into high expression and low expression according to a cutoff value of 30% of positively stained cells.

### Extraction and selection of radiomic features

After image acquisition, PET and CT images in the DICOM format were imported into LIFEx freeware (v7.0.0 https://www.lifexsoft.org/), which automatically fused them for quantitative PET/CT analysis [21]. Two experienced nuclear medicine physicians, each with over 15 years of diagnostic experience, manually segmented the three-dimensional volume of interest (VOI) on every slice of primary breast lesions and metastatic lymph nodes. They were blinded to the clinical and pathological outcomes, except for the presence of BC. The VOI was defined with a threshold of 40% of the maximum standardized uptake value (SUV_max_), which included MTV and total lesion glycolysis (TLG). Intensity discretization for CT data was performed with the number of gray levels of 400 bins and absolute scale bounds from − 1,000 to 3,000 HU. In contrast, it was segmented into 64 bins and measured with SUV_max_ ranging from 0 to 20 for PET images. Moreover, the radiomic features of PET and CT were calculated automatically by this software for texture analysis. Intra-class correlation coefficients (ICCs) were calculated to evaluate the repeatability of all the features, of which ICCs > 0.75 were selected for model construction. Subsequently, the least absolute shrinkage and selection operator (LASSO) regression algorithm and t-test were used for the selection of the remaining features. Finally, 10-fold cross-validation was used to ensure the robustness of the optimal features.

### Construction and evaluation of the prediction model

Univariate regression analysis was used to screen out clinicopathological risk factors with statistically significant differences from potential predictors, including age, BMI, menopausal status, tumor location, histological types, clinical TNM staging, and molecular types. The above-mentioned risk factors were subsequently combined with radiomic features and analyzed by multivariable logistic regression analysis to develop a prediction model. The receiver operating characteristic (ROC) curve, Hosmer-Lemeshow (H-L) test, and decision curve analysis (DCA) were used to evaluate the discrimination, calibration, and clinical usefulness of the model, respectively.

### Statistical analysis

**Fig. 1:**
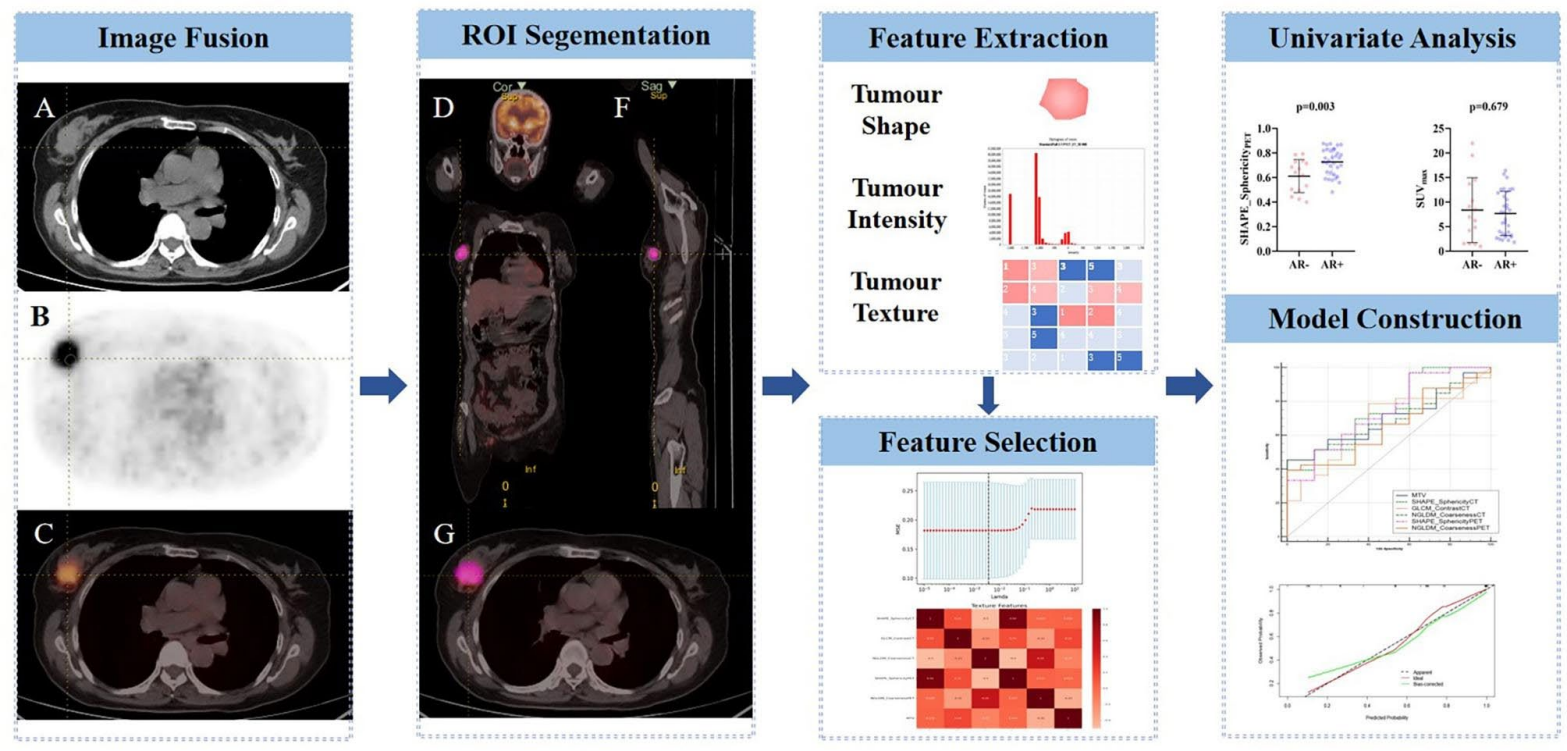
The workflow of this study. CT image (**A**), PET image (**B**), PET/CT fusion image (**D**), coronary image (**E**), sagittal image (**F**), and transaxial image (**G**) of ^18^F-FDG PET/CT display an example of VOI for extracting imaging features of BC. A patient with stage IV of BC underwent PET/CT image showing a metabolically active right breast lesion

All statistical data were calculated and analyzed with IBM SPSS statistics version 26.0, Python version 3.11 (https://www.python.org), MedCalc software (MedCalc Software, Ostend, Belgium), and R version 4.2.1 (http://www.R-project.org). The normality and homogeneity of variance of continuous data were evaluated by the Kolmogorov-Smirnov test and Levene’s test, respectively. The numeric variables were tested by the independent t-test and Mann-Whitney U test. Instead, the Chi-square test and Fisher’s exact test were applied to analyze categorical variables. P < 0.05 was considered statistically significant. Figure 1 presents the workflow of this study.

## Results

### Clinicopathologic characteristics

A total of 48 BC patients were included in our study. Table 1 shows the baseline characteristics of these patients. Among the 48 patients, 33 patients (68.75%) were histologically confirmed as AR+, while 15 patients (31.25%) were confirmed as AR-. In our univariate analysis, MTV was tightly relevant to the expression of AR (p = 0.032). The ER and PR status were weakly correlated with AR status (p = 0.127 and 0.061, respectively). However, there was no significant difference found across the other clinical characteristics between the AR + and AR- groups.

**Table 1:** Characteristics of included BC patients with different AR expression statuses

Characteristics	AR- (n = 15)	AR+ (n = 33)	t/x^2^/z	p-value
Age (years)	56.00 ± 15.28	53.70 ± 13.74	0.520	0.606
BMI (kg/m^2^)	24.69 ± 4.25	24.82 ± 4.02	0.100	0.921
Menopausal Status			0.162	0.688
Premenopausal	5(33.3%)	13(39.4%)		
Postmenopausal	10(66.7%)	20(60.6%)		
Tumor Location			0.014	0.907
Left	8(53.3%)	17(51.5%)		
Right	7(46.7%)	16(48.5%)		
Histologic Type			0.514	0.662
IDC	12(80.0%)	29(87.9%)		
Other	3(20.0%)	4(12.1%)		
Clinical Stage			0.730	0.393
I-II	4(26.7%)	13(39.4%)		
III-IV	11(73.3%)	20(60.6%)		
Clinical T Stage			0.183	0.738
T1-T2	10(66.7%)	24(72.7%)		
T3-T4	5(33.3%)	9(27.3%)		
Clinical N Stage			1.302	0.629
N0	4(26.7%)	14(42.4%)		
N1-2	6(40.0%)	9(27.3%)		
N3	5(33.3%)	10(30.3%)		
Clinical M Stage			0.032	1.000
M0	11(73.3%)	25(75.8%)		
M1	4(26.7%)	8(24.2%)		
ER Status			2.334	0.127
Positive	7(46.7%)	23(69.7%)		
Negative	8(53.3%)	10(30.3%)		
PR Status			3.499	0.061
Positive	3(20.0%)	16(48.5%)		
Negative	12(80.0%)	17(51.5%)		
HER2 Status			0.162	0.688
Positive	5(33.3%)	13(39.4%)		
Negative	10(66.7%)	20(60.6%)		
Molecular Subtype			3.232	0.337
HR+/HER2-	6(40.0%)	15(45.5%)		
HR+/HER2+	1(6.7%)	8(24.2%)		
HER2+	4(26.7%)	5(15.2%)		
TNBC	4(26.7%)	5(15.2%)		
Ki-67			0.041	0.839
<30%	5(33.3%)	12(36.4%)		
≥30%	10(66.7%)	21(63.6%)		
SUV_max_	8.38 ± 6.61	7.70 ± 4.49	0.416	0.679
SUV_mean_	2.66 ± 2.02	2,94 ± 1.91	-0.477	0.636
TLG*	62.00(19.11 ~ 126.26)	44.26(15.62 ~ 129.46)	-0.612	0.541
MTV*	35.50(20.83 ~ 64.00)	17.70 (8.70 ~ 47.53)	-2.146	0.032

*Those data are described as median (lower quartile ~ upper quartile) and tested by the Mann-Whitney U test. AR-, androgen receptor negative; AR+, androgen receptor positive; t: independent t-test; x^2^, Chi-square test; z, Mann-Whitney U test; BMI: body mass index; ER, estrogen receptor; PR, progesterone receptor; HER2, human epidermal growth factor receptor-2; IDC, invasive ductal breast carcinoma; HR, hormone receptor (included ER and/or PR); TNBC, triple-negative breast cancer; Ki-67, cellular proliferation index; SUV_max_, maximum standardized uptake value; SUV_mean_, mean standardized uptake value; TLG, total lesion glycolysis; MTV, metabolic tumor volume

### Feature selection

**Fig. 2:**
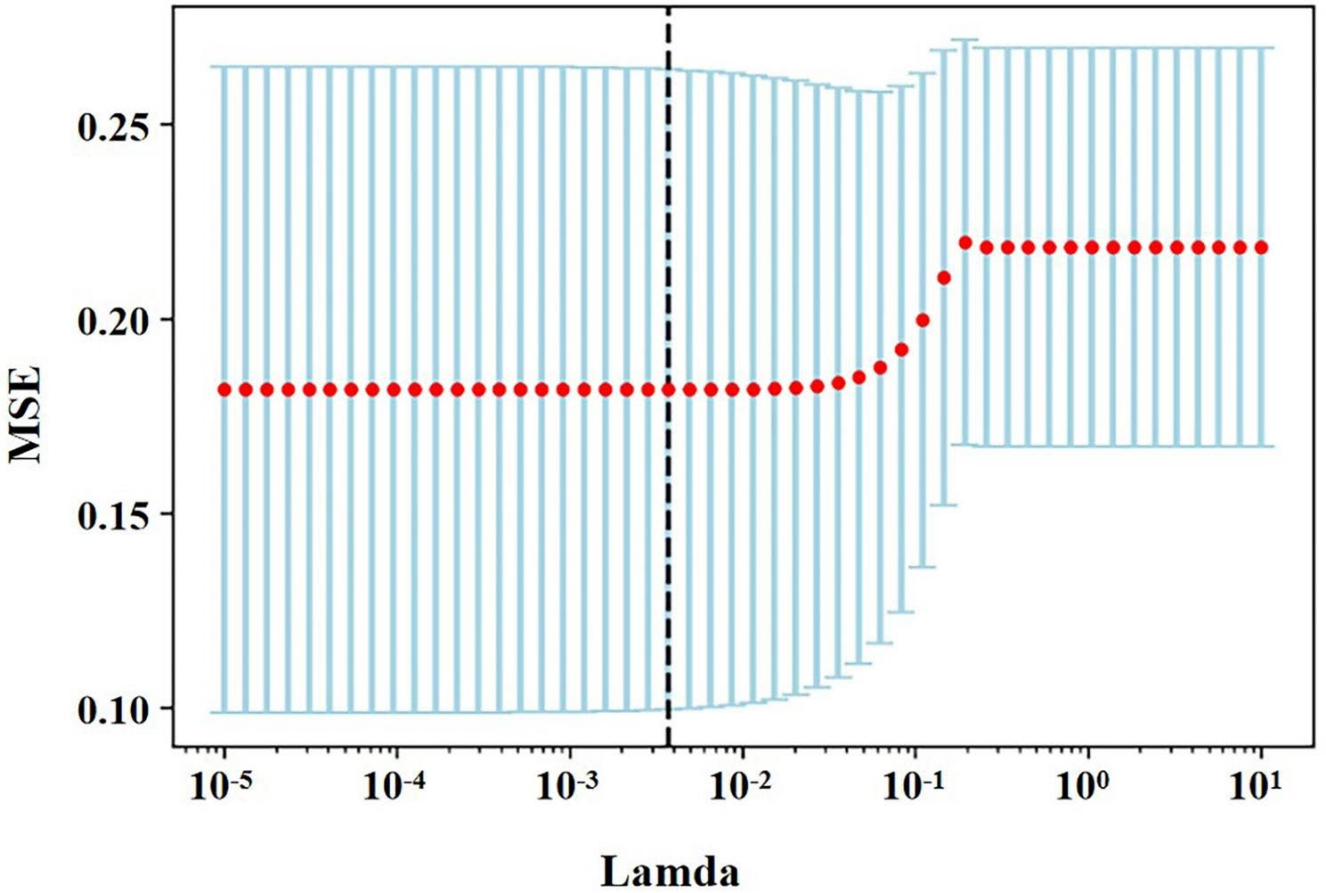
The Lamda of LASSO regression. The least absolute shrinkage and selection operator (LASSO) was conducted to select the radiomic features of CT and PET. Using 10-fold cross-validation, the suitable value of tuning parameter Lambda (λ) in LASSO regression was selected, and a vertical line was drawn here. MSE: mean squared error

**Fig. 3:**
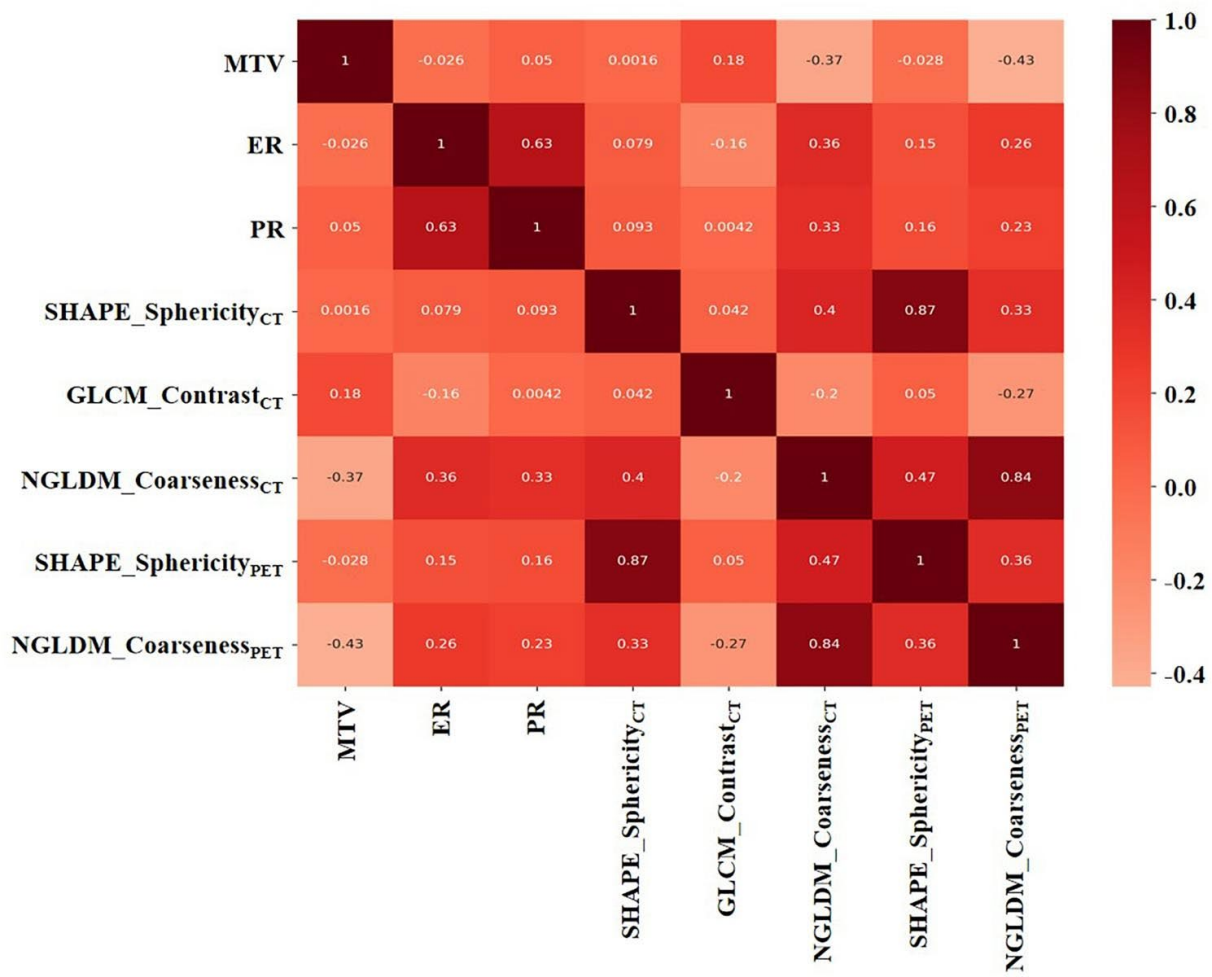
The Pearson correlation coefficient matrix heatmap in the prediction of AR expression. The darker color presents a higher correlation between the two factors

Table 2 presents 80 radiomic features extracted from CT and PET datasets. Five features were considered valuable for the prediction of the AR expression using both the t-test and LASSO regression model. Figure 2 shows the most suitable Lambda parameter of LASSO regression. For the CT dataset, SHAPE_Sphericity_CT_, GLCM_Contrast_CT_, and NGLDM_Coarseness_CT_ (CT Coarseness from neighborhood gray-level difference matrix) were selected. SHAPE_Sphericity_PET_ (PET Sphericity from SHAPE) and NGLDM_Coarseness_PET_ (PET Coarseness from neighborhood gray-level difference matrix) were extracted from the PET dataset. The ICC of the radiomic signatures was all above 0.75. Included MTV, we calculated the Pearson correlation coefficient between these features. Figure 3 displays the Pearson correlation coefficient matrix heatmap.

**Table 2:** Radiomic parameters extracted from PET/CT images

Conventional	First-order	Higher-order			
		GLZLM	GLRLM	GLCM	NGLDM
SUV/HU_min_	HISTO_Skewness	GLZLM_SZE (Short-Zone Emphasis)	GLRLM_SRE (Short-Run Emphasis)	GLCM_Homogeneity	NGLDM_Coarseness
SUV/HU_mean_	HISTO_Kurtosis	GLZLM_LZE (Long-Zone Emphasis)	GLRLM_LRE (Long-Run Emphasis)	GLCM_Energy	NGLDM_Contrast
SUV/HU_std_	HISTO_Entropy_log10	GLZLM_LGZE (Low Gray-level Zone Emphasis)	GLRLM_LGRE (Low Gray-level Run Emphasis)	GLCM_Contrast	NGLDM_Busyness
SUV/HU_max_	HISTO_Entropy_log2	GLZLM_HGZE (High Gray-level Zone Emphasis)	GLRLM_HGRE (High Gray-level Run Emphasis)	GLCM_Correlation	
SUV_peak_*	HISTO_Energy	GLZLM_SZLGE (Short-Zone Low Gray-level Emphasis)	GLRLM_SRLGE (Short-Run Low Gray-level Emphasis)	GLCM_Entropy_log10	
TLG*	SHAPE_Sphericity	GLZLM_SZHGE (Short-Zone High Gray-level Emphasis)	GLRLM_SRHGE (Short-Run High Gray-level Emphasis)	GLCM_Entropy_log2	
	SHAPE_Compacity	GLZLM_LZLGE (Long-Zone Low Gray-level Emphasis)	GLRLM_LRLGE (Long-Run Low Gray-level Emphasis)	GLCM_Dissimilarity	
	SHAPE_Volume (mL)	GLZLM_LZHGE (Long-Zone High Gray-level Emphasis)	GLRLM_LRHGE (Long-Run High Gray-level Emphasis)		
	SHAPE_Volume(vx)	GLZLM_GLNU (Gray-Level Non-Uniformity for zone)	GLRLM_GLNU (Gray-Level Non-Uniformity for run)		
		GLZLM_ZLNU (Zone Length Non-Uniformity)	GLRLM_RLNU (Run Length Non-Uniformity)		
		GLZLM_ZP (Zone%)	GLRLM_RP (Run Percentage)		

*Calculated only for the PET data. SUV, standardized uptake value; TLG, total lesion glycolysis; HISTO, Histogram; GLZLM, Gray-Level Zone Length Matrix; GLRLM, Gray-Level Run Length Matrix; GLCM, Gray-Level Co-Occurrence Matrix; NGLDM, Neighboring Gray-level dependence matrix

### Model construction

**Fig. 4:**
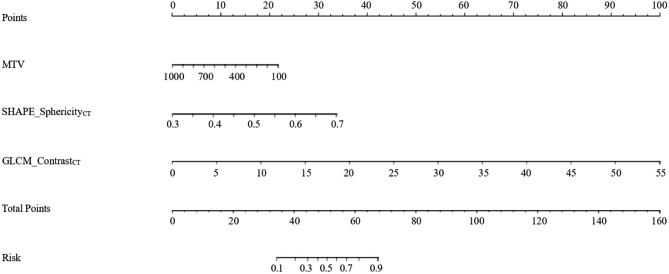
The nomogram of risk factors predicting the expression of AR. The importance of each variable that established the diagnostic model was visualized as the points

**Fig. 5:**
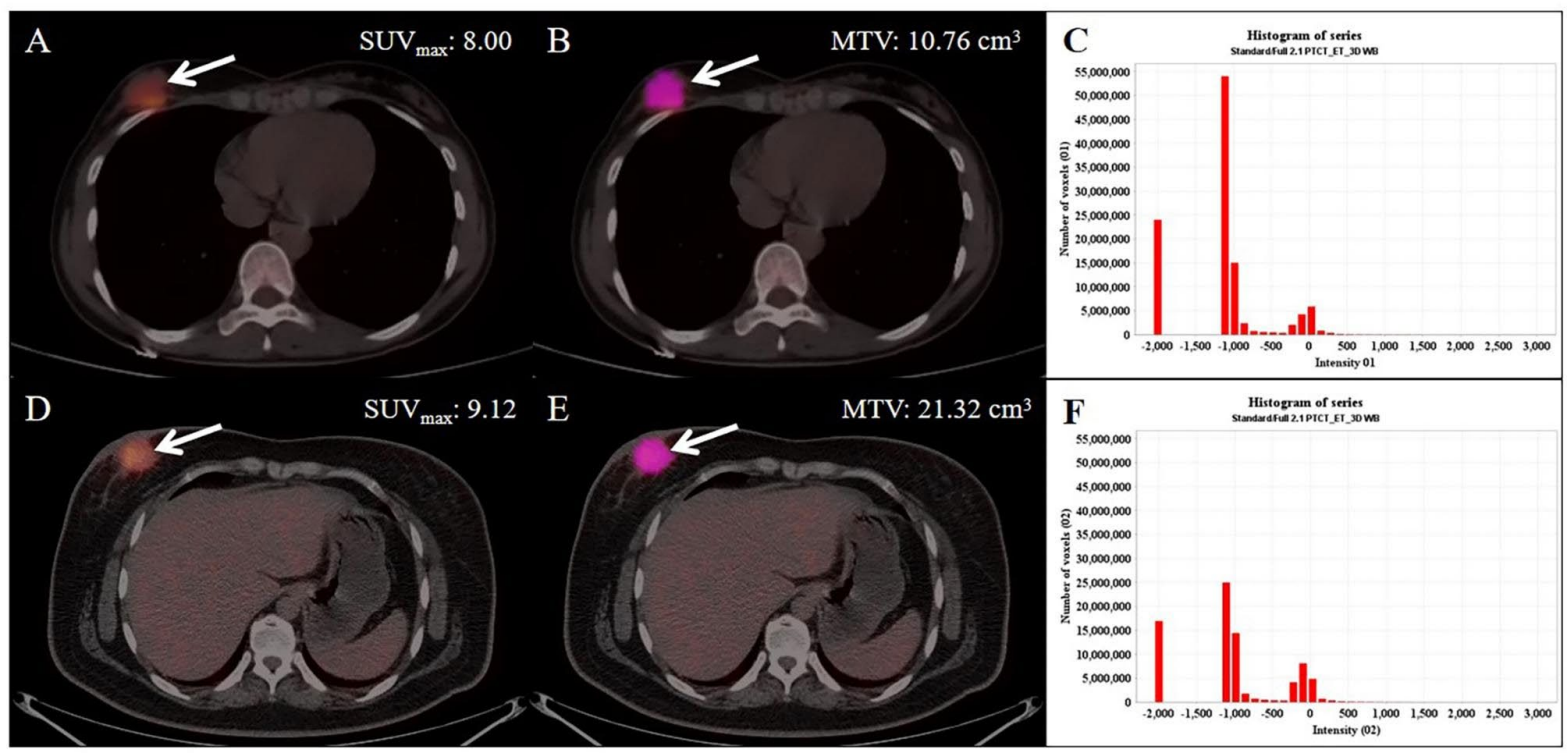
A typical case of the relationship between MTV and AR expression. Two patients were diagnosed with IDC (invasive ductal breast carcinoma) successively in a year. The former (**A**: PET/CT fusion image, **B**: The VOI of the lesion, **C**: The histogram of VOI) was confirmed as AR-positive (SUV_max_: 8.00; MTV: 10.76 cm^3^). The latter (**D**: PET/CT fusion image, **E**: The VOI of the lesion, **F**: The histogram of VOI) was confirmed as AR negative (SUV_max_: 9.12; MTV: 21.32 cm^3^). These lesions were similar on PET/CT images but showed significant differences in the histograms of the radiomic features

In the univariate analysis, the potential meaningful factors were selected for further multivariable regression analysis (p < 0.20) [22]. In order to prevent the omission of possible associated factors, those with P < 0.15 were included in our multivariate analysis. In addition, ER and PR have been confirmed to be correlated with AR expression in other research, and thus they were also included in our multivariable analysis [23]. Based on the above risk factors, we developed a nomogram capable of predicting the AR expression (Fig. 4). According to the cutoff of the ROC curve, continuous variables were transformed into binary variables (Table 3). It was worth mentioning that high MTV might be related to the negative expression of AR (Fig. 5). Moreover, these lesions were similar on PET/CT images but showed significant differences in the histograms of the radiomic features. Based on binary logistic regression, a diagnostic model consisting of significant risk factors was constructed, including MTV, SHAPE_Sphericity_CT_, and GLCM_Contrast_CT_ (Table 4). GLCM_Contrast_CT_ with a significant statistical difference between the AR + and AR- (OR [odds ratio]: 1.420; 95% CI [confidence interval]: 9.000 [1.460–55.478]; P = 0.018) groups was the independent predictive factor by using the Forward stepwise regression method.

**Table 3:** ROC analyses for meaningful risk factors

Features	AUC (95% CI)	cutoff	p	Se (%)	Sp (%)
SHAPE_Sphericity_CT_	0.747 (0.601–0.862)	> 0.6152	0.0010*	39.39	100.00
GLCM_Contrast_CT_	0.673 (0.522–0.801)	> 7.3826	0.0346*	78.79	60.00
NGLDM_Coarseness_CT_	0.691 (0.541–0.816)	> 0.0020	0.0113*	45.45	100.00
SHAPE_Sphericity_PET_	0.739 (0.593–0.855)	> 0.7362	0.0017*	51.52	86.67
NGLDM_Coarseness_PET_	0.653 (0.501–0.784)	> 0.0431	0.0587	39.39	100.00
MTV	0.695 (0.545–0.820)	≤ 13.1048	0.0096*	45.45	100.00

ROC, receiver operating characteristic; AUC, the area under the curve; Se, Sensitivity; Sp, Specificity. *p < 0.05

**Table 4:** The Univariable and multivariate analysis of this prediction model

Variable	Uni-p	Multi-p	OR (95%CI)
MTV ≤ 13.1048	0.002*	0.998	
ER status	0.127		
PR status	0.061		
SHAPE_Sphericity_CT_ > 0.6152	0.004*	0.998	
GLCM_Contrast_CT_ > 7.3826	0.008*	0.018*	9.000 (1.460-55.478)
NGLDM_Coarseness_CT_ > 0.0020	0.008*		
SHAPE_Sphericity_PET_ > 0.7362	0.012*		
NGLDM_Coarseness_PET_ > 0.0431	0.004*		

^*^p < 0.05. Previous continuous variables were transformed into binary variables and then analyzed by the Chi-square test in the Univariable analysis. OR (odds ratio) with its 95% CI was used to estimate correlation strength

### Model effectiveness evaluation

**Fig. 6:**
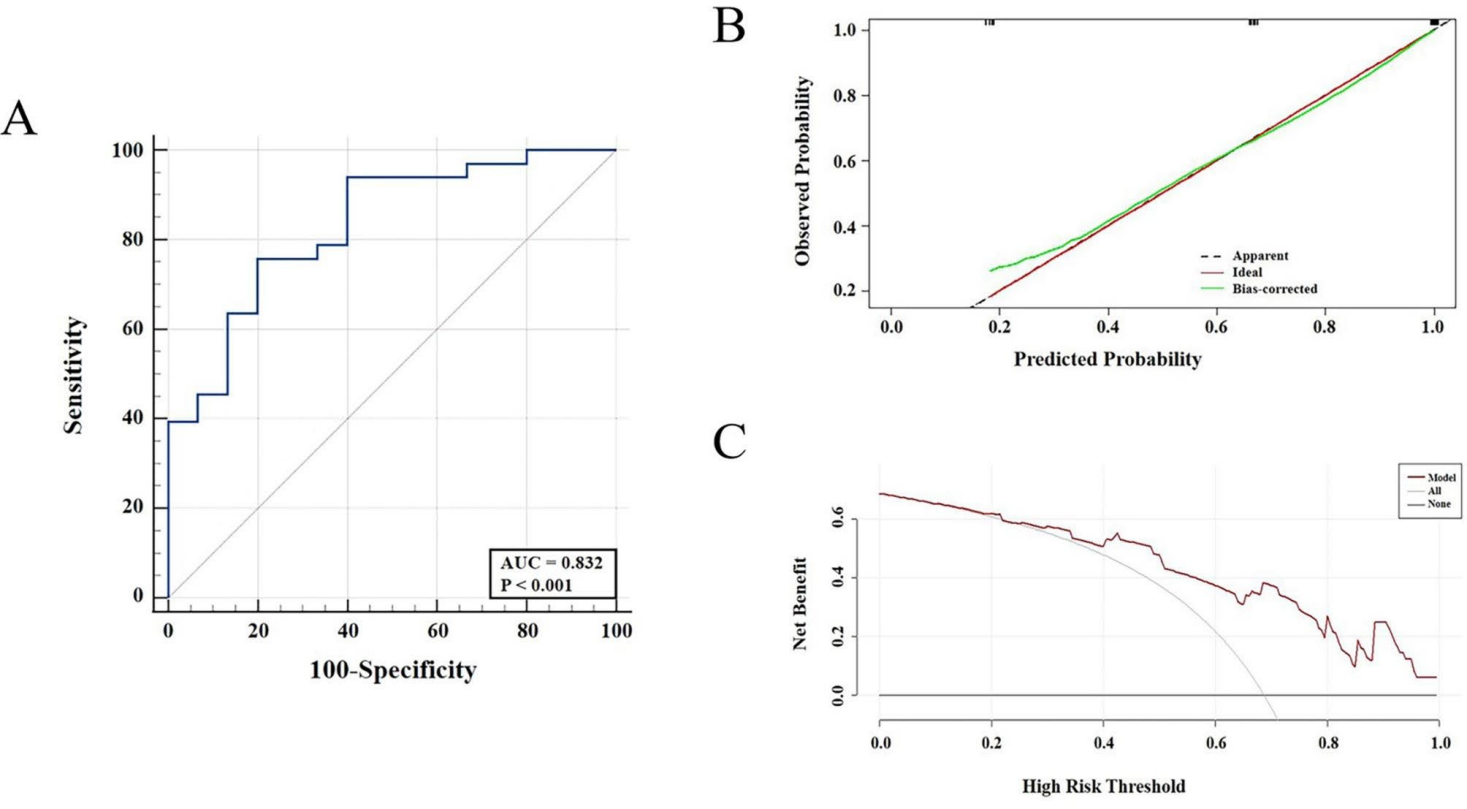
The evaluation of the prediction model of AR expression in internal validation using Bootstrap. The ROC curve of the combined model consisted of radiomic features and clinical characters (**A**); the calibration curve (green) after correction of bias fluctuates around the ideal curve (red), which revealed the good accuracy between the actual probability and predicted probability (**B**); decision curve showed that the prediction model led to a higher net benefit than intervention or no-intervention of all patients among a large range of threshold (**C**)

Figure 6 A shows the ROC curve of the combined prediction model. The AUC of the combined model for predicting the AR + was 0.832 (95% CI: 0.697–0.924). The sensitivity and specificity were 75.76% and 80.0%, respectively. Subsequently, 10-fold cross-validation was used to verify the stability of the AUC. The mean value of AUC was 0.833. After bootstrap resampling, we got the calibration curve (Fig. 6B). The concordance index (c-index) of this calibration curve was 0.885 (95%CI: 0.805–0.965), which was reduced to 0.882 after the bias correction. The p-value of the H-L test was beyond 0.05. These results all provided a higher calibration of this model. Finally, the DCA of this study displayed that more benefits could be added to clinical strategies when this prediction model was used to predict the AR expression (Fig. 6C).

## Discussion

Some studies have focused on the correlation between the status of other receptors and the extensive expression of AR, indicating that it can promote the proliferation of cancer cells [24]. Accumulating preclinical and clinical evidence supports that some patients can benefit from AR inhibitor therapy, which especially improves the prognosis of TNBC patients [25–27]. In order to avoid the limitations of IHC, recent studies have demonstrated the feasibility of radiomics combined with clinical data for predicting intrinsic molecular subtypes and the expressions of receptors in BC [28, 29]. However, only very few studies have focused on predicting the expression of AR status. In our present study, MTV, SHAPE_Sphericity_CT_, GLCM_Contrast_CT_ extracted from ^18^F-FDG PET/CT could predict the AR status in BC patients. We further developed a machine-learning model with a higher AUC score on the ROC curve (0.832, 95% CI: 0.697–0.924).

Previous studies have shown that standard parameters (SUV_max_, SUV_mean_, and MTV) extracted from PET images are associated with ER or PR status [17, 30, 31]. In our univariate analysis, there was no obvious correlation between SUV_max_, SUV_mean_, and AR status. Huang et al. have concluded that ER and PR status have significant differences between AR + and AR- groups [23]. ER and PR status were weakly correlated with the expression of AR (p = 0.061, p = 0.127) in our study. The reason was attributed to the residual confounding factors, and it needs to be further investigated in a larger cohort population [32, 33]. However, high MTV showed a tendency to be associated with AR negativity (p = 0.032). Kaida et al. have also found a similar situation, in which a feeble correlation exists between high MTV and ER-negative status [31]. In further textural analysis, SHAPE_Sphericity_CT_ (p = 0.004), GLCM_Contrast_CT_ (p = 0.008), NGLDM_Coarseness_CT_ (p = 0.008), SHAPE_Sphericity_PET_ (p = 0.012), and NGLDM_Coarseness_PET_ (p = 0.004) selected by LASSO and t-test were correlated with the expression of AR. In multivariable logistic analysis, only SHAPE_Sphericity_CT_ and GLCM_Contrast_CT_ were included in the prediction model. Due to the limited density resolution of the PET images, they performed worse than CT images in screening and extracting meaningful radiomics signatures [34]. Currently, GLCM (grey-level co-occurrence matrix) textural features are used to classify the tumor grade of BC [35–38]. Here, we are the first to report the independent performance of GLCM_Contrast_CT_ in predicting AR molecular markers (p = 0.018).

Based on the forward stepwise logistic regression, we established a combined model, in which the accuracy was usually assessed by discrimination, calibration, and clinical applicability [39, 40]. According to its AUC (AUC = 0.832), this model presented a better efficacy in the discrimination of AR status. Cross-validation and Bootstrap have proved to be used for the predictive performance of a small sample [41]. Furthermore, using the above methods for internal validation, we finally obtained a very stable value of AUC (AUC = 0.833). Some studies have proved that the c-index, with its 95% CI, can provide a more comprehensive assessment of calibration [42, 43]. Combined with the p-value of the H-L test, our model showed a higher capacity for stability [44]. DCA can be used to map the utility of a net benefit assessment model in decision-making within a clinically reasonable risk threshold [45]. The DCA curve of this study visualized that the diagnostic model achieved better net benefit than the all-intervention or no-intervention strategy within a larger threshold range, further proving the excellent clinical applicability of this model.

To the best of our knowledge, we originally established a combined model to predict AR expression based on ^18^F-FDG PET/CT imaging. There were some limitations in our present study due to the single-center and retrospective design. Next, due to the novelty of the molecular receptor detection of AR, the number of patients included in this study was relatively small. Thirdly, except for internal validation, the resulting model needed to be validated with an external cohort to improve the confidence of clinical applicability. Lastly, a classical supervised classification algorithm should be used to construct a prediction model, and deep learning-based features from PET/CT images need to be verified in further studies.

## Conclusions

In conclusion, by combining clinicopathological factors and radiomic features extracted from ^18^F-FDG PET/CT images, we established a model to predict AR expression in BC patients with a single primary lesion. Our model could serve as a novel strategy to select patients who could benefit from anti-AR treatment and to assist clinicians in making clinical decisions.

## Data Availability

The original contributions presented in the study are included in the article. Further inquiries can be directed to the corresponding authors.
